# Protective effects of metformin in various cardiovascular diseases: Clinical evidence and AMPK‐dependent mechanisms

**DOI:** 10.1111/jcmm.17519

**Published:** 2022-09-02

**Authors:** Yizhi Bu, Mei Peng, Xinyi Tang, Xu Xu, Yifeng Wu, Alex F. Chen, Xiaoping Yang

**Affiliations:** ^1^ Key Laboratory of Study and Discovery of Small Targeted Molecules of Hunan Province, Key Laboratory of Protein Chemistry and Developmental Biology of Fish of Ministry of Education, Department of Pharmacy School of Medicine, Hunan Normal University Changsha Hunan China; ^2^ Institute for Developmental and Regenerative Cardiovascular Medicine, Xinhua Hospital, School of Medicine, Shanghai Jiao Tong University Shanghai China

**Keywords:** cardiovascular diseases, metformin, protective effect

## Abstract

Metformin, a well‐known AMPK agonist, has been widely used as the first‐line drug for treating type 2 diabetes. There had been a significant concern regarding the use of metformin in people with cardiovascular diseases (CVDs) due to its potential lactic acidosis side effect. Currently growing clinical and preclinical evidence indicates that metformin can lower the incidence of cardiovascular events in diabetic patients or even non‐diabetic patients beyond its hypoglycaemic effects. The underlying mechanisms of cardiovascular benefits of metformin largely involve the cellular energy sensor, AMPK, of which activation corrects endothelial dysfunction, reduces oxidative stress and improves inflammatory response. In this minireview, we summarized the clinical evidence of metformin benefits in several widely studied cardiovascular diseases, such as atherosclerosis, ischaemic/reperfusion injury and arrhythmia, both in patients with or without diabetes. Meanwhile, we highlighted the potential AMPK‐dependent mechanisms in in vitro and/or in vivo models.

## 1 INTRODUCTION

Cardiovascular disease (CVD) is one of the leading causes of mortality globally, of which incidence has continuously increased per year since 1990. In 2019, CVD caused 18.6 million deaths with a ratio of approximately 30% of deaths in the world and was prevalent among approximately 6% of the global population with total 523 million people.^1^ CVDs are a series of circulatory diseases caused by genetic and environmental factors, including hypertension and obesity. Specially, diabetes is considered as a major risk factor in the development of CVD.^2^ Common diseases of cardiovascular system include atherosclerosis (AS), ischaemia–reperfusion injury and myocardial fibrosis. The occurrence and development of these diseases are the results of complicated interactions in the body, involving cell death,^3^ inflammation, oxidative stress and immune response.^4^

Metformin, a well‐known AMP‐activated protein kinase (AMPK) agonist, has been widely used for the treatment of type 2 diabetes mellitus (T2DM) over 50 years.^5^ Due to its low toxicity, high efficacy and affordable cost, metformin continued to be certified as the first‐line drug for treating T2DM.^6^ Mechanistically, metformin directly inhibits mitochondrial complex I of the electron transport chain,^7^ leading to increased AMP/ATP ratio and canonical AMPK activation. Once activated by energetic stress, AMPK mediates a wide range of downstream signalling pathways, including peroxisome proliferator‐activated receptor gamma coactivator 1‐alpha (PGC‐1α), mammalian target of rapamycin (mTOR) and endothelial NOS (eNOS).^8^ In spite of the existence of AMPK‐independent action of metformin, it has been shown that metformin exerts beneficial effects mainly in an AMPK‐dependent manner.^9^

Cardiovascular protective effects of metformin were firstly discovered by UK Prospective Diabetes Study Group in 1998. This multicentre trial recruited 1704 overweight patients with T2DM to investigate whether there is any advantage between conventional group and intensive glucose control with metformin, chlorpropamide, glibenclamide and insulin, respectively.^10^ After a median follow‐up of 10.7 years, metformin‐treated group (maximum dose = 2550 mg daily) showed a 30% lower risk of all macrovascular diseases than the conventional group and the lowest incidence of macrovascular and microvascular complications compared with all other drug‐treated groups.^10^ Profoundly, the similar improved HbA_1c_ levels in all intensive groups were observed, indicating that the protective cardiovascular effects of metformin are independent of its glycaemic control. Thus, this trial greatly stimulated the interest of scientists to explore the cardio‐protective effects of metformin both clinically and mechanistically.^10^ After this trial, Holman et al conducted a 10‐year post‐trial monitoring to further seek whether such intensive metformin treatment has a long‐term effect on macrovascular outcomes. They observed significantly continuous risk reductions in any diabetes‐related end point and myocardial infarction in metformin‐treated group.^10^, ^11^ Since then, numerous clinical studies have conducted to evaluate effects of metformin on the cardiovascular system in patients with or without diabetes, and a summary of related clinical trials or meta‐analysis is shown in Table 1.

**TABLE 1:** Summary of included clinical trials and meta‐analysis regarding the effects of metformin on cardiovascular system

Type of trial	Patients	Intervention and group	Major findings
Multicentre trial^10^	1704 overweight patients with T2DM	1 Conventional group 2 Intensive blood‐glucose control: 1 Chlorpropamide; 2 Glibenclamide; 3 Insulin; 4 Metformin (XL 2550 mg daily)	Metformin group showed the lowest incidence of macrovascular and microvascular complications in all groups, lower risk of all‐cause mortality and macrovascular diseases than the conventional group.
10‐year post‐trial monitoring^11^	3277 patients with T2DM		Above risk reductions were sustained throughout the post‐trial period.
Multicentre, randomized, double‐blind, placebo‐controlled clinical trial^12^	304 T2DM patients with coronary artery disease (CAD)	1. Glipizide (30 mg daily) for 3 years 2. Metformin (1.5 g daily) for 3 years	Metformin group showed a significant reduction in composite cardiovascular end points (death from a cardiovascular cause, death from any cause, nonfatal myocardial infarction, nonfatal stroke, arterial revascularization) than glipizide group, but no significant difference about the secondary end points (new or worsening heart failure, new critical cardiac arrhythmia, new or worsening angina).
Meta‐analysis^13^	Patients with T2DM		Cardiovascular mortality in metformin group was lower versus sulfonylureas.
Randomized controlled trial^14^	4038 patients with T2DM and chronic kidney disease (CKD)	1. Metformin users 2. Non‐metformin users	Metformin may be safer for use in CKD than previously considered and may lower the risk of death and cardiovascular events in individuals with stage 3 CKD.
Multicentre double‐blind, randomized, placebo‐controlled trial^15^	428 adults with T1DM at increased risk for CVDs	1. Metformin (1000 mg twice daily) for 3 years 2. Placebo for 3 years	Metformin did not change mean cIMT, endothelial function and insulin dose, but reduced maximal cIMT, bodyweight, LDL cholesterol, indicating that metformin might have a wider role in cardiovascular risk management.
Randomized, double‐blind, placebo‐controlled study^16^	33 non‐diabetic women with angina and normal coronary arteries	1. Metformin (500 mg twice daily) for 8 weeks 2. Placebo	Metformin may improve endothelium‐dependent microvascular responses and decrease myocardial ischaemia.
Controlled trial^17^	60 patients with MS and without diabetes and structural cardiac diseases	1. Metformin (850 mg daily) for 1 year + dietary counselling 2. Dietary counselling	Metformin has considerable beneficial effect on nitroxidation, endothelial function, inflammation and cIMT in patients with MS.
Multicentre prospective study^18^	258 prediabetic patients with stable angina and nonobstructive coronary artery stenosis	1. Patients with normoglycaemia 2. Patients with prediabetes (pre‐DM) 3. Patients with prediabetes treated with metformin (850 mg twice daily)	Metformin therapy may reduce the high risk of cardiovascular events in pre‐DM patients by reducing coronary endothelial dysfunction.
Pilot double‐blind, placebo‐controlled trial^19^	18 children with obesity and risk markers for metabolic syndrome (MS)	1. Metformin (850 mg/day) for 24 months 2. Placebo	Metformin is efficacious, well tolerated, and has potential long‐term benefits in the improvement of body composition and inflammation markers in such patients.
Randomized controlled trial^20^	68 non‐diabetic patients with coronary artery disease, with insulin resistance and/or prediabetes	1. Metformin (XL 2000 mg daily dose) for 12 months 2. Placebo	Metformin decreased LVMI, LVM, office systolic blood pressure, body weight and markers of oxidative stress, supporting the cardio‐protective role of metformin.
Double‐blind, randomized controlled trial^21^	173 non‐diabetic patients with coronary heart disease	1. Metformin (850 mg twice daily) 2. Placebo	Metformin had no effect on cIMT and little or no effect on several surrogate markers of cardiovascular disease in non‐diabetic patients with high cardiovascular risk, taking statins.
Randomized, placebo‐controlled trial^22^	390 patients with T2DM	1. Insulin+metformin (maximally 850 mg daily) for 16 weeks 2. Insulin+placebo	Metformin treatment improved endothelial function, but not chronic, low‐grade inflammation in patients with type 2 diabetes treated with insulin.
Randomized double‐blind, placebo‐controlled, cross‐over trial^23^	41 statin‐treated obese patients with coronary artery disease and newly diagnosed T2DM	1. Liraglutide+metformin for 4 weeks 2. Placebo+metformin for 4 weeks (0.5–1.0 g, twice daily)	Liraglutide combined with metformin may improve the atherogenic LDL lipid profile and CRP.
Prospective, double‐blind randomized clinical study^24^	40 T1DM patients	1. Empagliflozin (25 mg daily) for 12 weeks 2. Metformin (2000 mg daily) for 12 weeks 3. Empagliflozin/metformin (25 mg daily and 2000 mg daily) for 12 weeks 4. Placebo	Metformin improved endothelial function but did not affect arterial stiffness.
Randomized controlled trial^25^	90 children with T1DM	1. Metformin (maximally 1 g twice a day) for 12 weeks 2. Placebo	Metformin improved vascular smooth muscle function and HbA1c, and lowered insulin dose in type 1 diabetes children, but had no effect on other cardiovascular risk factors (such as fat mass, lipid profile and CRP).
Double‐blind Randomized controlled trial^26^	42 patients with carotid artery atherosclerosis	1. Metformin (500 mg twice a day) for 12 weeks 2. Placebo	Metformin ameliorates the pro‐inflammatory state in patients with carotid artery atherosclerosis through Sirtuin 1 induction.
Randomized clinical trial^27^	3234 patients with prediabetes and early diabetes mellitus	1. Metformin (850 mg twice daily) with an average duration of 14 years 2. Placebo 3. Lifestyle modification	Metformin may protect against coronary atherosclerosis by decreasing CAC severity and presence in prediabetes and early diabetes among men.
Single‐centre, open‐label phase II trial^28^	20 patients with idiopathic or heritable PAH	Metformin(2 g/day) for 8 weeks	Metformin therapy was safe and well tolerated in patients with PAH, and may be associated with improved RV fractional area change and reduced RV triglyceride content.
Prospective, randomized study^29^, ^30^	93 patients with PAH associated with congenital heart defects (CHD)	1. Bosentan (initially at 62.5 mg twice daily for 4 weeks and then 125 mg twice daily) for 3 months 2. Metformin (500 mg twice daily) for 3 months 3. Metformin+Bosentan for 3 months	Combination therapy with bosentan and metformin in PAH‐CHD patients improved exercise capacity and pulmonary haemodynamics, compared with bosentan alone. Additionally, in vitro decreased pulmonary artery contraction is possibly related to increased AMPK phosphorylation.
Randomized, placebo‐controlled trial^31^	390 T2DM patients treated with insulin	1. Metformin+insulin (850 mg, 1–3 times daily) for 4 months 2. Placebo+insulin	Metformin reduced the risk of macrovascular disease (including myocardial infarction, heart failure, acute coronary syndrome and transient ischaemic attack).
Pilot, randomized trial^32^	152 metabolic syndrome patients following percutaneous coronary intervention (PCI) (with no prior history of metformin treatment)	1. Metformin (250 mg of 3 times a day), for 7 days before PCI 2. Control group	Metformin pretreatment regimen significantly reduces postprocedural myocardial injury and improves 1‐year clinical outcomes in metabolic syndrome patients undergoing PCI.
Double‐blind, placebo‐controlled study^33^, ^34^	380 non‐diabetic patients presenting with MI and undergoing primary PCI	1. Metformin (500 mg, twice daily) for 4 months 2. Placebo	The use of metformin compared with placebo did not result in improved left ventricular ejection fraction and did not exert beneficial long‐term effects, which do not support the use of metformin in this setting.
Retrospective propensity score‐matched cohort study^35^	390 patients with T2DM and hypertension	A follow‐up of 6 years	Metformin improved diastolic function and delayed the progression of HFpEF in patients with T2DM and hypertension, had a lower risk of developing MACEs.
Population‐based dynamic cohort and in vitro studies^36^	645,710 patients with T2DM, not using other anti‐diabetic medication	1. Metformin users 2. Nonusers	Metformin decreased risk of AF in patients with T2DM who were not using other anti‐diabetic medication, probably via attenuation of atrial cell tachycardia‐induced myolysis and oxidative stress.
Meta‐analysis^37^	271 patients with DM and AF after catheter ablation (CA)	1. Metformin users 2. Nonusers	Metformin appears to be independently associated with a significant reduction in the risk of recurrent atrial arrhythmias after CA for AF.

In this minireview, we particularly focus on summarizing the cardio‐protective effects of metformin. The recent evidence of metformin on cardiovascular events in both clinical settings and experimental models is collected, and the updated knowledge about AMPK‐dependent molecular mechanisms involved is highlighted.

## 2 EFFECTS OF METFORMIN IN VASCULAR SYSTEM DISEASES

### 2.1 Clinical evidence

A placebo‐controlled trial examined whether metformin achieves cardio‐protective effects through improving endothelial function and lowering inflammation in 390 T2DM patients treated with insulin. Patients in metformin‐treated group underwent 16‐week treatment and a 4‐year follow‐up, whose blood samples were observed to have the reduced markers of endothelial dysfunction, but no significant changes in markers of low‐grade inflammation compared to placebo group. We notice that since only two biomarkers of inflammation, C‐reactive protein (CRP) and soluble intercellular adhesion molecule‐1 (sICAM‐1) were determined in this trial; thus, it is not clear whether metformin decreased inflammatory activity or not.^22^ In another placebo‐controlled, cross‐over trial in statin treated obese patients with T2DM and coronary artery disease, no change of CRP level was observed, but TNFα decreased in metformin alone group, implying that anti‐inflammatory activity of metformin was more related to the regulation of TNFα but not CRP in T2DM patients, consistent with previous trial. Additionally, atherogenic LDL lipid profile was also improved, indicating a positive effect of metformin on atherogenic processes.^23^

Remarkably, the REMOVAL study (REducing with MetfOrmin Vascular Adverse Lesions) initiated by Petrie et al for type 1 diabetes mellitus (T1DM) patients has been the largest and longest trial to evaluate effects of metformin on vascular function in T1DM. In this multicentre and double‐blind trial, metformin treatment lasted 3 years and more than 400 adults with T1DM at increased risk for cardiovascular disease were recruited. The primary endpoint was the averaged mean of far‐wall carotid artery intima‐media thickness (cIMT, a surrogate of atherosclerosis), and secondary endpoints included a range of biomarkers reflecting metabolism and endothelial function. Interestingly, metformin treatment did not reduce the progression of mean cIMT but significantly decreased the maximal cIMT, a better reflective of atherosclerosis progression than mean cIMT.^15^, ^25^ However, there was no improvement of endothelial function measured by reactive hyperaemia index (RHI), which seems to conflict with another trial that found improved endothelial function after 3‐month treatment of metformin.^24^ Besides in adults with T1DM, effects of metformin on vascular function were also evaluated in children (8–18 years).^25^ In this 12‐month randomized controlled trial, vascular smooth muscle function was represented by flow‐mediated dilatation and glyceryl trinitrate‐mediated dilatation (GTN).^25^ Compared to placebo, metformin augmented GTN level indicating metformin could improve vascular function in T1DM children.^25^ These trials in T1DM population revealed the promising but undefined benefit of metformin on T1DM patients' vascular system.

The Diabetes Prevention Program Outcomes Study (DPPOS) examined effects of metformin on the development of atherosclerosis using coronary artery calcium (CAC) measurements in patients with diabetes or prediabetes. Surprisingly, with an average duration of 14 years, this double‐blinded and randomized trial firstly found that metformin significantly lowered CAC severity among men, but no beneficial effect was seen in women. The more evident effects of metformin on CAC were found in younger men with lower CAC scores, rather than older men with more severe CAC, suggesting that metformin may be more efficacious on early stages of atherosclerosis in men.^27^

A multicentre prospective study assessed effects of metformin in patients with prediabetes with stable angina and nonobstructive coronary stenosis.^18^ After 2‐year follow‐up, it was found that metformin decreased the markers of inflammation and oxidative stress, with appearance of improved epicardial endothelial dysfunction, thus lowering the incidence of major adverse cardiac events (cardiac death, myocardial infarction and heart failure).^18^ Ameliorated inflammatory state presented by reduction of CRP, IL‐6 and TNFα was also observed in patients with carotid artery atherosclerosis after 3‐month metformin treatment.^26^ In patients with metabolic syndrome (MS), metformin also displayed the protective effects on vascular system.^17^, ^19^ Involving 60 patients with metabolic syndrome in the absence of diabetes and structural cardiac diseases, a controlled trial identified that 1‐year metformin treatment could reduce IMT and CRP levels, which was also demonstrated in obese children with MS,^19^ indicating a better vascular function and alleviated inflammatory response.^17^ Furthermore, it was interesting that nitroxidative stress was significantly improved, predicting the higher NO availability and better endothelial function after metformin treatment.^17^

Several clinical trials supported metformin's beneficial role in pulmonary arterial hypertension (PAH). A single‐arm trial involving 20 patients with idiopathic or heritable PAH discovered that metformin improved the structure and lipid metabolism of right ventricular without any apparent adverse effects, indicating that metformin therapy was safe and well tolerated in patients with PAH.^28^ A prospective, randomized study investigated the efficacy of a combination of Bosentan, an endothelin receptor antagonist with metformin in 93 PAH patients associated with congenital heart defects. The results demonstrated that addition of metformin had greater improved pulmonary haemodynamics compared to Bosentan monotherapy after 3‐month treatment.^30^ Further, increased AMPK and eNOS phosphorylation was discovered in in vitro pulmonary artery contraction induced by phenylephrine after combination therapy, indicating the pivotal role of AMPK in metformin‐induced beneficial effects.^29^, ^30^

However, the CAMERA study (Metformin for non‐diabetic patients with coronary heart disease), a randomized controlled trial, challenged metformin's purported cardiovascular benefits. It was found that metformin treatment for 18 months had neither effect on cIMT and little nor effect on inflammatory state and lipid profile. A possible explanation for this is that all participants in CAMERA were taking statins already, then limited the ability of metformin to show a reduction in cardiovascular risks. Thus, whether metformin can be recommended for treating this population needs further well‐designed clinical trial.^21^

Taken these results together, most of clinical trials in the vascular system mainly demonstrated the protective effect of metformin in atherosclerosis and PAH. Metformin exhibits therapeutic benefit in vascular system through improving endothelial dysfunction, reducing oxidative stress, and inhibiting inflammatory response and lipid accumulation. However, it seems like that the efficacy of metformin highly depends on the baseline characteristics of the patients, such as the type of diabetes, sex, age, administration of other lipid‐lowering drugs and different study endpoints. Therefore, further evidence is needed to figure out which types of patients are suitable for getting vascular benefits from metformin.

### 2.2 AMPK‐related actions of metformin in atherosclerosis

**FIGURE 1:**
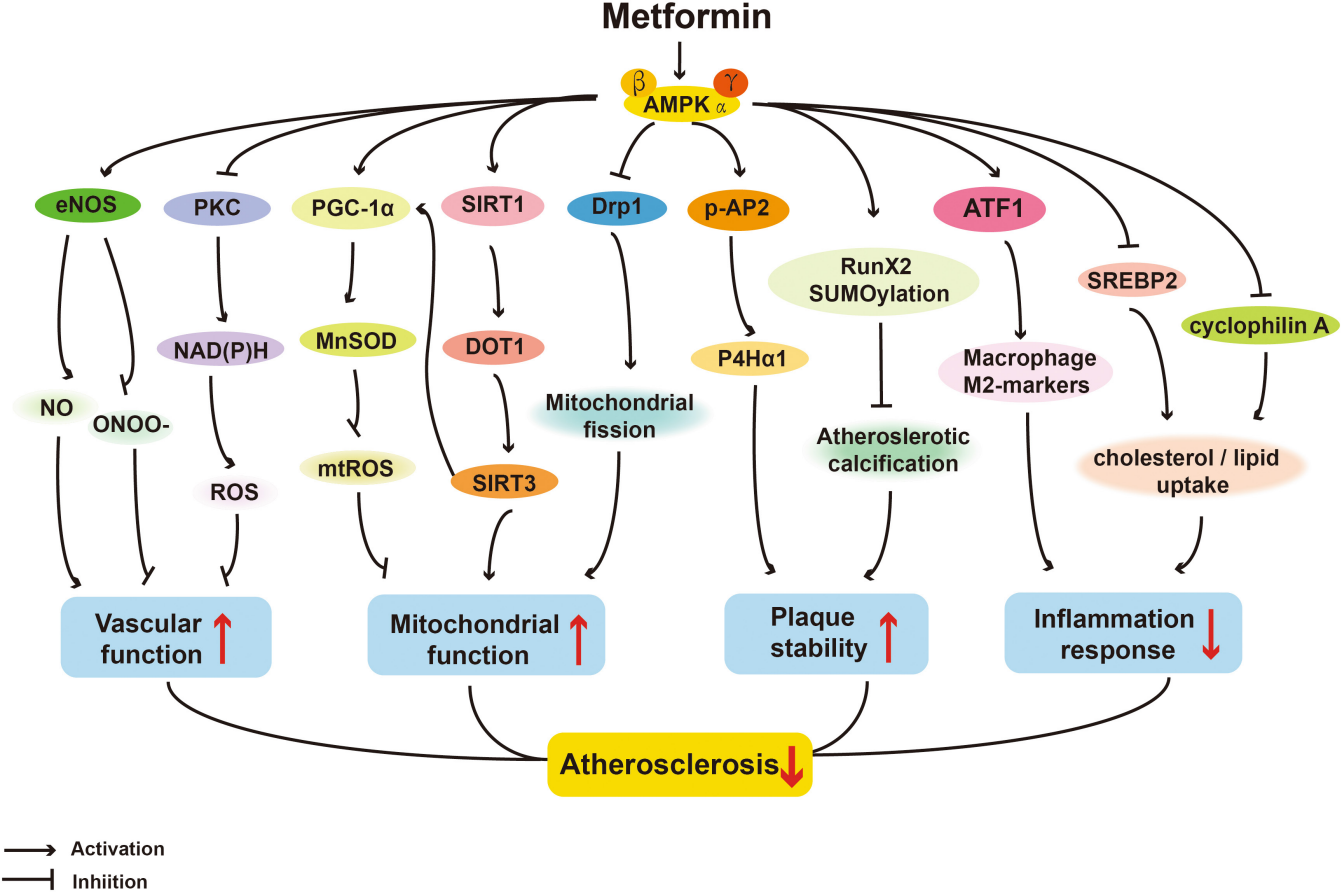
AMPK‐dependent actions of protective effects of metformin on atherosclerosis. eNOS, endothelial nitric oxide synthase; NO, nitric oxide; PKC, protein kinase C; PGC‐1α, peroxisome proliferator‐activated receptor gamma coactivator‐1α; MnSOD, manganese superoxide dismutase; mtROS, mitochondrial reactive oxygen species; SIRT1, sirtuin‐1; DOT1, disruptor of telomeric silencing‐1 like protein or Kmt4; SIRT3, sirtuin‐3; Drp1, dynamin‐related protein; p‐AP2, phosphorylation of activator protein 2 alpha; P4Hα1, prolyl‐4‐hydroxylase alpha 1; Runx2, Runt‐related transcription factor 2; SUMO, small ubiquitin‐like modifier; ATF1, activating transcription factor 1; SREBP2, Sterol regulatory element‐binding protein 2.

Activation of AMPK could be a potential strategy for treating AS through improving endothelial dysfunction and mitochondrial dysfunction, reducing oxidative stress and inflammatory responses.^38^, ^39^ In line with these actions, metformin has been reported to delay the development of AS from these aspects in an AMPK‐dependent manner (Figure 1).

In diabetic mice, metformin improved endothelial function via inhibiting endoplasmic reticulum (ER) stress and increasing NO bioavailability. The underlying mechanism is related to activation of AMPK/peroxisome proliferator‐activated receptor δ (PPARδ) pathway.^40^ Metformin exerted its vasculoprotective effects related to suppressing mitochondrial fission^41^ and strengthening mitochondrial biogenesis in an AMPK‐dependent manner,^42^ regarding mitochondrial homeostasis. Furthermore, several studies provided valid rational to utilize metformin as a stabilizer of atherosclerotic plaque.^43^, ^44^ In diabetic ApoE^−/−^ mice, chronic administration of metformin inhibited atherosclerotic plaque formation^42^ and markedly reduced atherosclerotic calcification,^43^ which were abolished by knockout of AMPK, indicating the critical role of AMPK in functioning the effect of metformin. Moreover, AMPKα inactivation was proved to destabilize atherosclerotic plaque, further identifying that the effect of metformin is in an AMPK‐dependent fashion.^44^ Profoundly, Yan et al and You et al,^45^, ^46^ respectively, demonstrated that the proliferation and migration of vascular smooth muscle cells (VSMCs) and HUVECs are the major composition of atherosclerotic plaques while metformin could suppress these proliferation and migration through activating different AMPK‐dependent pathways, alleviating diabetes‐accelerated AS.

The occurrence of atherosclerosis was closely related to vascular oxidative stress caused by overproduction of ROS.^47^ In above‐mentioned clinical trials, metformin showed the ability to inhibit oxidative stress in blood vessels.^17^, ^18^ To our knowledge, two ways of intracellular ROS production are either PKC‐dependent activation of NAD(P)H oxidase on cell membrane or the increase of mitochondrial superoxide production.^48^ In ECs, the action of metformin's inhibitory effect on intracellular ROS production was linked with these two pathways, particularly inhibiting NAD(P)H oxidase by activating AMPK.^47^, ^49^ In mitochondria, metformin was able to increase MnSOD (manganese superoxide dismutase) expression to normalize ROS production and enhance mitochondrial biogenesis via the activation of AMPK‐peroxisome proliferator‐activated receptor gamma coactivator‐1α (PGC‐1α) pathway.^50^

The eNOS pathways have been shown to get involved in oxidative stress in ECs. Thus, dysfunction of eNOS leads to disordered vasodilatation.^51^, ^52^ According to this mechanism, metformin was shown to enhance the endothelium‐dependent vasorelaxation via increasing eNOS in T2DM rat,^53^ which was consistent with the results in patients with MS.^17^ However, the role of AMPK in mediating metformin to raise NO has not been clearly clarified yet and needs further investigation. On the one hand, metformin could inhibit vascular calcification through AMPK/eNOS/NO pathway in rat aortic smooth muscle cells.^51^ On the other hand, it was found that metformin could obviously reverse high glucose‐induced decrease in phosphorylation of eNOS with no alteration of AMPK phosphorylation.^52^ However, it was noteworthy to point out that after partial knockdown of total AMPK using siRNA, metformin was unable to reverse the decreased ratio of p‐eNOS/eNOS in mouse microvascular endothelial cells.^52^ These results implied that the function of metformin to upregulate eNOS expression is at least partially dependent on AMPK.

Active inflammation is an early precursor and a trigger of atherosclerotic changes.^54^ Metformin has been reported to exhibit anti‐inflammatory properties in animal models fed by atherogenic diet,^55^ providing the compelling evidence regarding the anti‐inflammatory effects of metformin in humans.^19^, ^26^ Furthermore, several studies elucidated the action of metformin on phenotypic transformation and lipid uptake of macrophages, which may be potential mechanisms of improvements of lipid profile in humans.^56^, ^57^, ^58^ Utilizing hyperlipidaemic mice and human macrophages, Seneviratne et al observed that metformin dramatically increased expression of the atheroprotective signature M2 genes depending on AMPK/ATF1 (activating transcription factor 1) pathway, indicating that metformin promoted the differentiation of macrophages to the M2 phenotype, an anti‐inflammatory state.^58^ Sterol regulatory element‐binding protein 2 (SREBP2), one of SREBP transcription factors, participates in lipid synthesis and cholesterol homeostasis.^56^, ^59^ It was found that metformin‐induced activation of AMPK could inhibit SREBP2, thereby improving lipid homeostasis in VSMCs and monocyte/macrophages.^56^ Cyclophilin A, a secreted protein with pro‐inflammatory properties in the vascular vessel wall, was also suppressed by metformin in an AMPK‐dependent fashion, reducing lipid uptake in macrophages and preventing transmigration and differentiation of monocytes.^57^ Collectively, these findings indicated that metformin worked on different stages of inflammatory cells through activating AMPK, from initial recruitment and adhesion of monocytes to the ultimate plaque formation by lipid‐laden foam cells.^55^

Obviously, above ex in vivo and in vitro studies suggest that metformin exhibits therapeutic benefit in AS by correcting endothelial dysfunction, inhibiting oxidative stress, suppressing inflammatory response and attenuating lipid accumulation.

### 2.3 AMPK‐related actions of metformin in PAH

Statements in previous section have shown the clinical evidence of metformin benefits on PAH; molecular mechanisms underlying have not been discussed in details yet. Among pathological mechanisms of PAH, vascular remodelling is extremely critical, wherein the proliferation of pulmonary arterial smooth muscle cells (PASMCs) is a critical contributor.^60^ Studies have shown that metformin could inhibit PASMCs proliferation in vitro and in ex vivo models; this effect was attenuated by AMPK knockout.^61^, ^62^, ^63^ Further, Liu et al and Zhai et al found that inhibition of autophagy was one of the underlying mechanisms, confirming the key function of AMPK activation in this anti‐proliferative effect.^64^, ^65^ Abnormal deposition of extracellular matrix (ECM) is another pathologic alteration resulting in vascular remodelling.^66^ In PAH rat models, AMPK activation by metformin inhibited ECM remodelling of pulmonary arteries, which was coupled with improved right ventricle structure.^66^

All of these findings in cultured cells and rat models revealed that metformin could prevent the AMPK activation‐dependent development of PAH, confirming by the clinical trial in PAH patients.^30^

## 3 EFFECTS OF METFORMIN IN HEART DISEASES

### 3.1 Clinical evidence

Metformin's benefit on heart diseases was found in the UK Prospective Diabetes Study Group, in which a 39% lower risk of myocardial infarction (MI) was observed in metformin group compared to conventional group.^10^ Two independent studies in T2DM patients further confirmed the association between metformin and lower risk of MI.^13^, ^31^ A meta‐analysis compared the effectiveness and safety of different hypoglycaemic drugs monotherapy, such as thiazolidinediones, metformin and sulfonylureas.^13^ It was noteworthy that a slightly lowered incidence of fatal MI was only observed in metformin group compared to other hypoglycaemic drug‐treated groups, implying the unique beneficial effect of metformin on cardiovascular system.^13^ ‘Hyperinsulinemia: the Outcome of its Metabolic Effects’ is a randomized, placebo‐controlled, multicentre trial to investigate long‐term effects of metformin in T2DM patients treated with insulin.^31^ Consistently, addition of metformin dramatically reduced the risk of MI and transient ischaemic attack after a 4‐month treatment and a 4.3‐year follow‐up, indicating that patients could benefit from sustained treatment of metformin and insulin.^31^ Additionally, several studies contributed to explore whether metformin could protect diabetic patients with diverse complications from MI.^12^, ^14^ David et al carried out a randomized controlled trial to investigate the safety and effects of metformin in patients with T2DM and chronic kidney disease (CKD). A decreased incidence of cardiovascular events, including MI, in metformin users with CKD stages G 1–3 but not CKD stage G 4–5 was found.^14^ Another trial in T2DM patients with coronary artery disease (CAD) examined the long‐term effects of glipizide and metformin on the major cardiovascular events.^12^ In a 3‐year treatment and a median 5‐year follow‐up, metformin substantially reduced the incidence of MI compared to glipizide, indicating a potential cardiovascular benefit of metformin therapy in such high‐risk patients.^12^ Percutaneous coronary intervention (PCI) is an established treatment strategy for coronary artery disease and associated with higher incidence of myocardial injury.^32^ A prospective and open‐label trial examined the effects of metformin pretreatment in MS patients scheduled for coronary intervention. In this trial, metformin pretreatment for 7 days significantly reduced the incidence of post‐PCI MI in 1 year, with observational results of low CRP levels and biomarkers of myocardial injury (creatine kinase‐MB and troponin I).^32^

However, in non‐diabetic patients, the effect of metformin remains debatable. In non‐diabetic women with angina and normal coronary arteries, 8‐week therapy with metformin decreased the frequency of exercise‐induced angina, implying a feasibly potential approach to improving myocardial ischaemia.^16^ However, to confirm this result, larger controlled trials of longer duration may be needed since this trial recruited only 33 patients.^16^ Another randomized clinical trial was conducted among 380 non‐diabetic patients presenting with ST‐segment elevation MI.^34^ Disappointedly, after 4‐month treatment with metformin and 2‐year follow‐up, neither the improvement of left ventricular function nor the decrease of major adverse cardiac events was observed, suggesting that metformin did not exert beneficial long‐term effects in this clinical setting.^33^, ^34^

The cardio‐protective effects of metformin were also reflected in inhibiting cardiac remodelling in diabetic or non‐diabetic patients. A single‐centre and retrospective study with 6‐year follow‐up focused on clinical data of long‐term metformin users with T2DM and hypertension but without symptoms of heart failure.^35^ This trial found that long‐term metformin exposure significantly reduced left ventricular (LV) mass index and the risk of new onset of symptomatic heart failure with preserved ejection fraction (HFpEF).^35^ Another randomized controlled trial in patients with coronary artery disease without diabetes also found that 1‐year treatment of metformin reduced left ventricular mass, which may be associated with alleviated oxidative stress.^20^ These two studies indicated that long‐term use of metformin could ameliorate cardiac remodelling regardless of the nature of myocardial insults or glycaemic status of patients.

Besides above clinical evidence on MI and cardiac remodelling, the effects of metformin on arrhythmia in humans have attracted considerable attention in recent years. Benefits of metformin on arrhythmia were firstly discovered in a retrospective analysis with the data of diabetic patients after acute MI from 1985 to 1993.^67^ In this clinical survey, patients treated with combination of metformin and other drugs experienced significantly less atrial fibrillation (AF) than patients who took no drugs or metformin alone.^67^ Then in 2014, Chang et al conducted the first population‐based epidemiological investigation on the association between metformin usage and development of AF.^36^ This study analysed approximately 9000 patients who newly diagnosed type 2 diabetes without using other anti‐diabetic drugs except metformin.^36^ After a 13‐year follow‐up, the significant decreased incidence of AF in metformin group was observed. However, this protective effects only sustained for 2 or 3 years during the follow‐up, indicating that metformin might be more efficient in the early stage of diabetes.^36^ Further, they demonstrated that benefit on AF was probably due to the reduction of both atrial cell lysis and ROS production.^36^, ^68^ As for patients with both T2DM and AF after catheter ablation (CA), metformin treatment (a median of 13 months after CA) also obviously reduced the risk of recurrent AF, but whether this effect was due to hypoglycaemic function or pleiotropic effects on electroanatomical mechanisms of AF remains unclear.^37^ Overall, current clinical evidence revealed the antiarrhythmic function of metformin in diabetic patients, but there has been no literature report on non‐diabetic patients so far.

### 3.2 Ex in vivo outcomes and AMPK‐related actions of metformin in ischaemic/reperfusion injury

To determine whether metformin ameliorates the cardiac structure and function on overall in I/R injury, researchers conducted animal studies using different dose of metformin and different duration of administration. As expected, Gundewar et al^69^ revealed the vital role of AMPK in metformin‐induced improvement of cardiac function in mice subjected to permanent left coronary artery (LCA). Metformin (125 μg/kg, i.p.) was given for 2 days before LCA ischaemic, then daily during reperfusion.^69^ The results showed that metformin treatment significantly increased survival rate by 47%, accompanied by up‐regulation of p‐AMPK, p‐eNOS and PGC‐1α expressions, which was abrogated in AMPK‐deficient mice, indicating the critical role of AMPK signalling.^69^ Yin et al implemented a longer‐term study in rats with large dose of metformin (250 mg/kg, po.), for 12 weeks.^70^ It was observed after administration of metformin, LCA ligation‐subjected rats had obvious smaller infarct size, improved left ventricular (LV) geometry and weakened LV remodelling.^70^ In addition, metformin also could exert cardio‐protective effects with an extremely short administration time in acute condition.^71^, ^72^ Furthermore, Palee et al observed that 200 mg/kg of metformin achieved the optimal effects on reducing infarct size compared to 100 and 400 mg/kg of metformin.^73^

In contrast, several conflicting observations of animal studies have been reported. In acute MI mouse models, administration of 100, 200 and 400 mg/kg of metformin for 1 week caused a significant increase rather than decrease in mortality of the MI mice in a dose‐dependent manner.^74^ In another study where a clinically relevant translational swine model was applied, metformin failed to reduce infarct size and to increase LV ejection fraction.^75^ Researchers analysed that the major reason may be due to that 1‐h metformin reperfusion duration time is too short, suggesting that the acute administration of metformin is probably not a viable option for clinical translation in humans.^75^ As we recall previous positive results in animal models, metformin is more likely to display its capability of reducing I/R injury with long‐term daily injection (at least 1 week) and a few days of pretreatment of metformin before ischaemia surgery. However, such protocols are almost unpractical in clinical settings because of the unpredictability of acute MI onsetting in non‐metformin long‐term users. Thus, in order to better reflect the effects of metformin in I/R injury, animal models closer mimicking clinic status as much as possible are needed in future.

**FIGURE 2:**
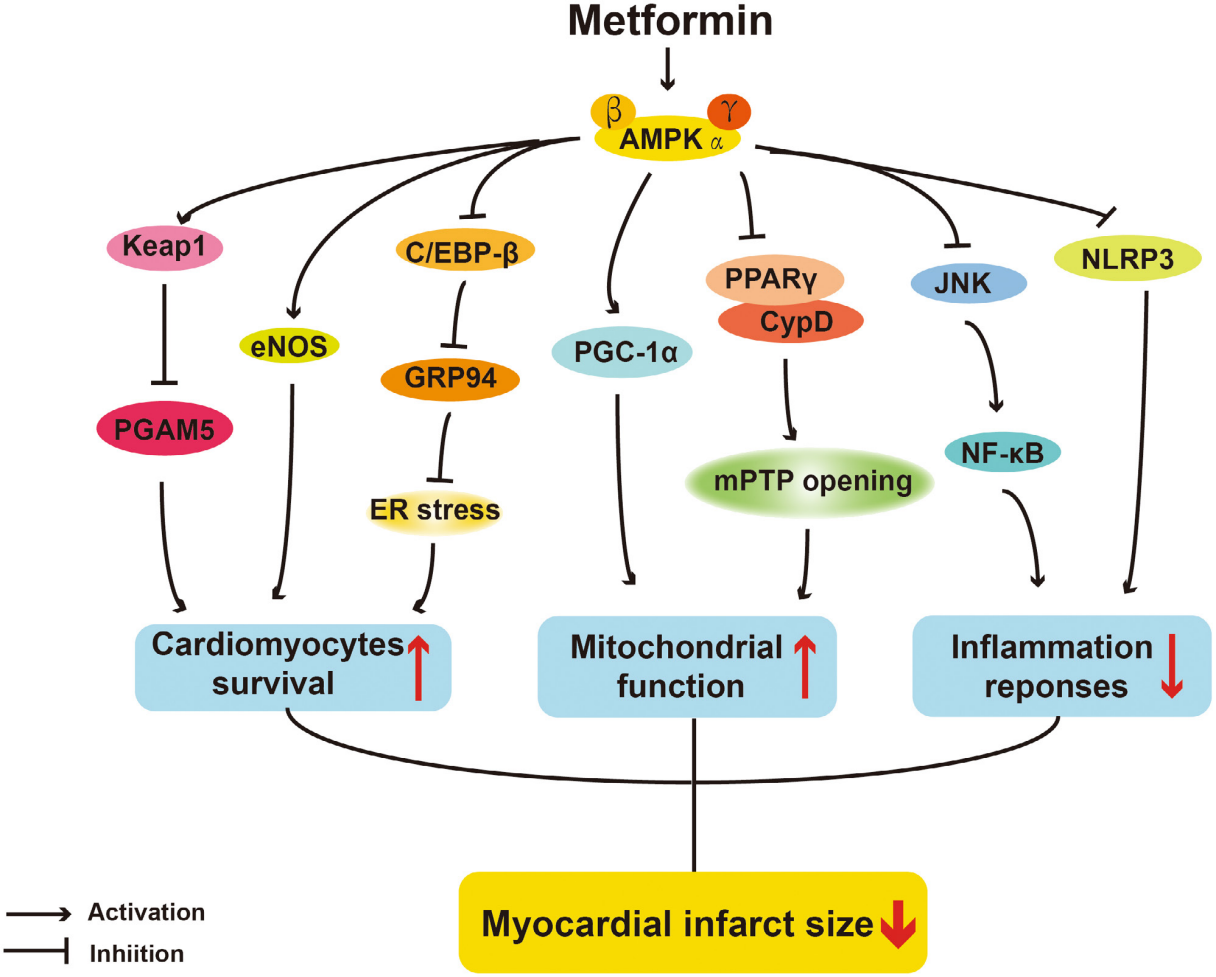
AMPK‐dependent actions of protective effects of metformin on I/R injury. PGAM5, phosphoglycerate mutase 5; C/EBP‐β, CCAAT/enhancer‐binding protein beta; GRP94, Hot shock protein 90b1; ER, endoplasmic reticulum; PGC‐1α, peroxisome proliferator‐activated receptor gamma coactivator‐1α; PPARγ, peroxisome proliferator‐activated receptor‐α; CypD, Cyclophilin D; JNK, c‐Jun N‐terminal kinase; NF‐κB, Nuclear factor kappa‐light‐chain‐enhancer of activated B cells; NLRP3, NOD‐like receptor protein 3.

Despite therapeutic effects of metformin in I/R injury animal models are not fully confirmed yet, the AMPK‐related molecular actions have been extensively studied (Figure 2). AMPK activation has been considered as a unique therapeutic potential for I/R injury via regulations of apoptosis, mitochondrial function and inflammatory response of cardiomyocytes.^76^ The anti‐apoptotic effect of metformin on cardiomyocytes was firstly revealed in an ex vivo experiment using dogs with pacing‐induced heart failure.^77^ This study found that metformin decreased H_2_O_2_‐induced apoptosis and improved cardiac function by promoting phosphorylation of both AMPK and eNOS.^77^ In damaged cardiomyocytes, post‐treatment with metformin attenuated cardiomyocytic apoptosis by lessening ER stress,^78^ in which mechanism underlying may be activation of AMPK then down‐regulation of ER chaperone proteins.^79^ For myocardial necroptosis, metformin also exhibited beneficial effects through AMPK activation. Phosphoglycerate mutase 5 (PGAM5) is a mitochondrial phosphatase required for programmed necrosis (necroptosis), and its ubiquitination degradation can be mediated by Keap1.^80^ Wang et al found that metformin could protect cardiomyocytes from several necroptosis inducers via promoting Keap1‐mediated PGAM5 degradation in the Langendorff‐perfused rat hearts, and this effect was abolished by AMPK inhibitor compound C, elucidating the important role of AMPK pathway.^80^

Mitochondrial dysfunction is one of the incentives of I/R injury, leading cardiomyocyte death. Through AMPK activation, metformin has been reported to improve mitochondrial function and activate some mitochondrial‐related biogenic molecules in cardiomyocytes. PGC‐1α is thought to serve as a vital regulator of mitochondrial biogenesis and function, and metformin could upregulate its expression by AMPK activation in I/R injury mice,^69^ then normalize shape and structure of mitochondria, and balance mitochondrial dynamics.^73^, ^81^ The mitochondrial permeability transition pore (mPTP) is a non‐specific channel on the mitochondrial inner membrane, whose opening will result in cardiomyocytes death in the first few minutes of myocardial reperfusion.^82^ The opening of mPTP is controlled by the Cyclophilin D (CypD), a master regulator of mitochondrial function.^83^ Barreto‐Torres et al identified that the interaction between the PPARα and CypD, which potentiated mPTP formation and opening, was abolished by metformin‐induced AMPK activation. Interestingly, changes of acetylation or phosphorylation of PPARα and CypD were not observed in metformin‐treated group in this study, denoting that metformin might induce their interaction through other types of post‐translational protein modifications.^84^

Inflammatory response is also an essential procedure causing myocardial tissue injury and cardiac remodelling, following to the release of many harmful pro‐inflammatory cytokines, such as TNF‐α, IL‐1β and IL‐6.^85^ In some of the above‐mentioned clinical trials, metformin indeed exhibited the ability to repress inflammatory responses.^32^ Several studies showed that metformin relieved inflammatory response in I/R injury through several different AMPK‐dependent pathways. Chen et al verified that metformin could inhibit mRNA levels of pro‐inflammatory cytokines TNF‐α and IL‐6 via inhibiting JNK‐NF‐κB signalling cascade in cardio‐myoblast cells, whereas knockdown of AMPKα abrogated these anti‐inflammatory effects of metformin, implying the critical role of AMPK pathway.^85^ NOD‐like receptor protein 3 (NLRP3) inflammasome is a significant component of the inflammatory immune response and plays an essential part in the development of I/R injury.^86^ Metformin could inhibit cellular apoptosis and inflammation by suppressing NLRP3 inflammasome via activating AMPK in neonatal rat ventricle myocytes (NRVMs) subjected to H/R.^87^

Principally, metformin treatment may also benefit stem cell transplantation, a therapeutic approach against MI, restoring lost cardiac function after I/R injury. Currently, two cell types, cardiosphere‐derived cells (CDCs) and mesenchymal stromal cells (MSCs), are promising to be applied in clinical settings.^88^ However, the poor survival of these stem cells in vivo seriously limits their therapeutic effects.^89^ In CDCs, Yue et al found that metformin attenuated CDC apoptosis and augmented its therapeutic effect, which might be associated with an AMPK‐eNOS‐dependent mechanism,^89^ implying that combination of CDCs with metformin is a promising strategy for MI patients. Unexpectedly, metformin in transplantation of MSCs had opposite effect. He et al found that metformin increased MSCs apoptosis by suppressing AMPK‐mediated mTOR and inhibiting other cell survival signalling, resulting in the impaired therapeutic efficacy of MSCs.^90^ Convincing interpretation for these paradoxical results may be the heterogeneity of the two cell types and the difference in these two protocols. Before LAD artery and transplantation of CDCs, metformin was injected 6 h prior to the surgery and CDCs were pretreated by metformin. However, before injecting MSCs, no pretreatment in animal body and infiltration in cells was implemented. The pretreatment of metformin before MI surgery seems to be indispensable. This explanation exactly coincides with our previous statement about conflicting outcomes in *ex* in vivo models that preconditioning is a key procedure for the beneficial effects of metformin in I/R injury. Thus, there is a risk for patients who accepted stem cell transplantation while taking metformin, and whether combination of stem cells with metformin could be extended to clinical practice requires further investigations.

### 3.3 AMPK‐related actions of metformin in cardiac remodelling

The key pathological features and main causes of cardiac remodelling are cardiomyocyte hypertrophy, fibrosis and cell death.^91^

Myocardial fibrosis is the excessive accumulation of ECM in cardiac tissue and its common feature is the differentiation of fibroblasts into myofibroblasts,^91^, ^92^ ultimately leading to cardiomyocyte cell death.^91^ This transition can be stimulated via mechanical stress, cytokines, growth factors and ECM components.^92^ In DCM rats, metformin down‐regulated several critical genes of fibrotic remodelling, such as ICAM, VCAM, COL‐I, COL‐III and TGF‐β.^93^ TGF‐β1 is the most potent activator mediating cardiac fibrosis, and the underlying mechanism of inhibition of TGF‐β1 by metformin was clarified in several studies.^94^, ^95^, ^96^ It has been explored that the TGF‐β1/ Smad pathway triggers pro‐fibrotic gene overexpression.^97^ Another independent study found that metformin inhibited TGF‐β1/Smad axis and reduced collagen synthesis in cardiac fibroblasts, thus attenuating cardiac fibrosis in mice subjected to TAC.^95^ Furthermore, this research team detected the role of AMPK in these inhibitory effects of metformin and found that Compound C did not reverse these protective effects while AICAR was disable to replicate them.^96^ A recent research using bioinformatics predictions from this group discovered another mechanism that TGF‐β1 can also be inhibited by the expression of suppressed hepatocyte nuclear factor 4α, which is regulated by metformin‐activating AMPK.^94^ Taken these three studies together, the direct inhibitory effects of metformin on TGF‐β1 depended on AMPK activation, but inhibition of the Smad signalling pathway was independent of AMPK activation, suggesting that metformin‐induced inhibition of TGFβ1 was triggered by both AMPK‐dependent and AMPK‐independent processes.

**FIGURE 3:**
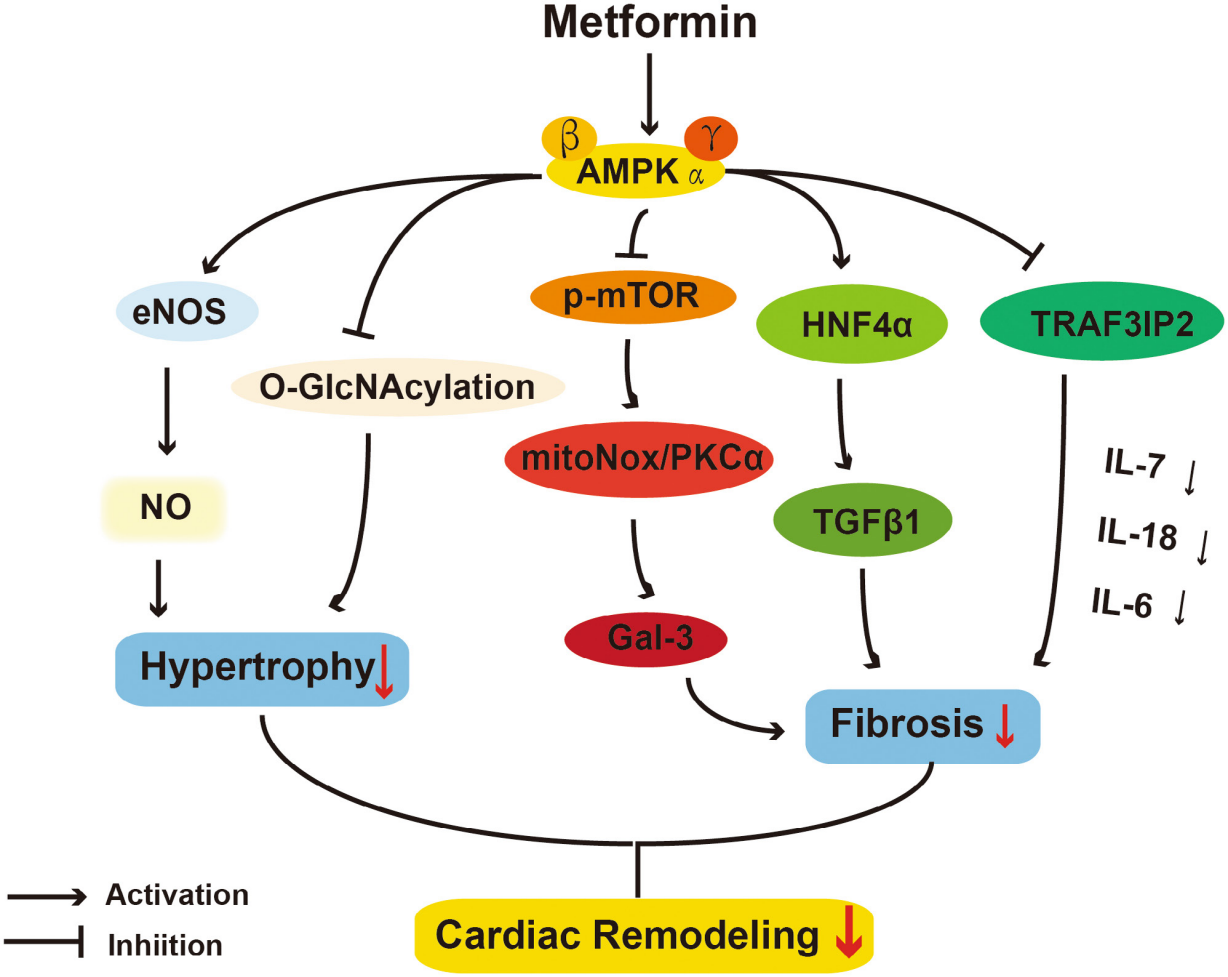
AMPK‐dependent actions of protective effects of metformin on cardiac remodelling. O‐GlcNAc, O‐linked N‐acetylglucosamine; p‐mTOR, phosphorylation of mammalian target of rapamycin; mitoNox, mitochondrial NADPH oxidase 4; Gal‐3, galectin 3; HNF4α, hepatocyte nuclear factor 4α; TGF‐β, transforming growth factor‐β; TRAF3IP2, TRAF3 Interacting Protein 2; IL, interleukin.

Oxidative stress is also an important factor that aggravates cardiac hypertrophy and fibrosis.^98^ Several studies provide important insights regarding antioxidant effects of metformin in experimental models. Overexpression of mitochondrial NADPH oxidase 4/PKC pathway, the well‐known oxidative stress‐related pathway, contributes to the development of adverse cardiac fibrosis. Up‐regulation of metformin‐induced AMPK inhibited this pathway and prevented the generation of matrix remodelling proteins, blocking the activation of the fibroblasts.^99^ Furthermore, oxidative stress could induce inflammation response through a series of molecular signal transduction. TRAF3 interacting protein 2 (TRAF3IP2) is an upstream regulator of some inflammatory mediators and an oxidative stress‐responsive adapter molecule.^100^ It was reported that the inhibitory effects of metformin on oxidative stress and inflammation were closely related to AMPK/TRAF3IP2 pathway, underlining the critical role of AMPK in metformin‐induced protection as well.^100^ All these studies highlight that the anti‐fibrotic effects of metformin are related to the inhibition of oxidative stress and inflammation (Figure 3).

The study regarding that metformin inhibits cardiac hypertrophy has made great progress (Figure 3). Transaortic contraction (TAC) is often used for establishment of hypertrophy models with significant haemodynamic dysfunction and ventricular hypertrophy.^101^ Zhang et al^101^ found that in TAC mice, metformin reduced cross‐sectional area of cardiomyocytes by about half and attenuated ventricular hypertrophy via activating AMPK/eNOS/NO pathway. Xu et al^102^ profoundly elucidated that metformin reduced TAC‐induced left ventricular hypertrophy through AMPKɑ2, but not AMPKɑ1. The different cellular and subcellular distribution of AMPKɑ1 and AMPKɑ2 may explain this interesting result. Specifically, AMPKɑ2 is mainly expressed in cardiac myosin, while AMPKɑ1 is more abundant in other cell types.^102^ This finding was also consistent with Gélinas R's conclusion that metformin treatment of myocardial hypertrophy mainly depended on AMPKɑ2‐dependent inhibition of protein O‐GlcNAcylation, an increased protein in hypertrophy condition.^103^

### 3.4 AMPK‐related actions of metformin in arrhythmia

Arrhythmia is reflected in ion channel disorders and the alternation of cardiac action potential duration.^104^ It is well‐known that AMPK is a powerful regulator of transport that regulates diverse ion channels and pumps on the cell membrane. Thus, AMPK has been considered as a critical modulator in cardiac electrophysiology and arrhythmia.^105^ In a swine I/R model, Lu et al^106^ found that the mortality rate of ventricular fibrillation in pigs treated chronically with metformin was 38% lower than those pigs without metformin treatment. The mechanism was presumed to be that metformin amplified the activation of AMPK in ischaemia and maintained myocardial ATP concentration. In addition, they found that acute metformin treatment has no effect on p‐AMPK, action potential dynamics or ventricular fibrillation in this model.^106^ These results are consistent with another observation that the acute metformin treatment affected neither the incubation period nor the mortality rate.^107^

Gap junction channels are necessary for propagating the action potential among adjacent cardiomyocytes.^108^ Abnormal expression of connexin 43 (Cx43) causes gap junction remodelling and cardiac arrhythmias.^109^ Interaction of Cx43 with zona occludens‐1 (ZO‐1) is also important for maintaining the normal width of gap junctions.^109^ In chronic atrial fibrillation (AF) model, gap junctions were down‐regulated and Cx43 was significantly decreased, then treatment of metformin could reverse this change by increasing the expression of ZO‐1 and promoting the interaction of Cx43 with ZO‐1, alleviating the vulnerability of AF.^109^ Additionally, these effects of metformin could be abolished by silencing AMPK, indicating the involvement of AMPK pathway. It was also found that metformin up‐regulated the level of Cx43 via inhibiting miR‐1 expression.^110^ Thus, inhibition of miR‐1 was likely associated with metformin‐mediated AMPK activation and C/EBP β reduction, based on the evidence that AMPK activator could decrease C/EBP β level and C/EBP β promoted miR‐1 expression directly.^79^

**FIGURE 4:**
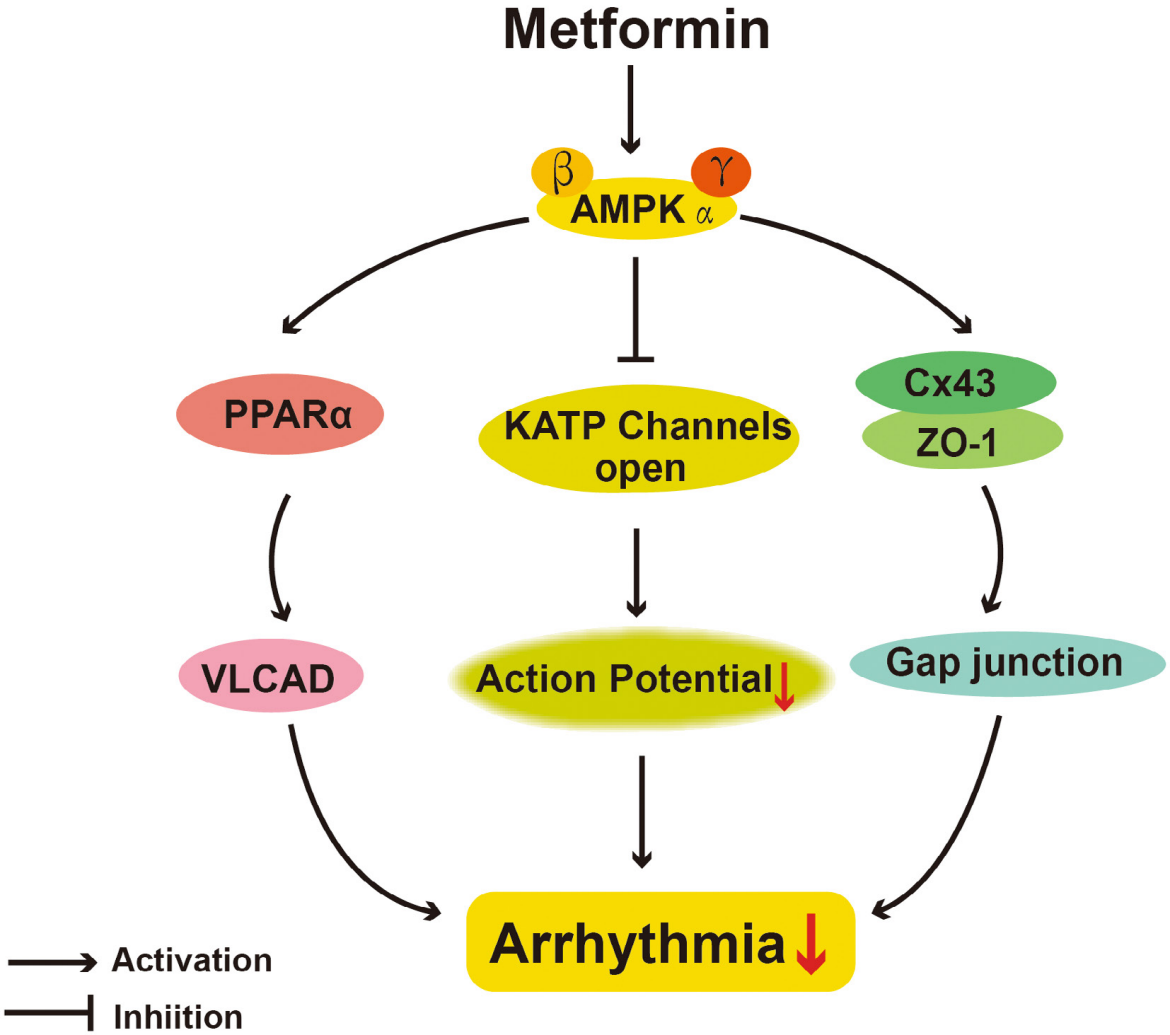
AMPK‐dependent actions of protective effects of metformin on arrhythmia. VLCAD, very‐long chain acyl‐CoA dehydrogenase; Cx43, connexin 43; ZO‐1, zona occludens‐1.

Recent work has highlighted the importance of abnormal fatty acid (FA) metabolism in cardiomyocytes in AF pathophysiology.^111^ Very‐long chain ACYL‐COA dehydrogenase (VLCAD) is the initial rate‐limiting enzyme in mitochondrial FA β‐oxidation, which is obviously decreased in canine models of AF. Bai et al found that metformin could affect FA β‐oxidation, then reduce fat accumulation through up‐regulating the AMPK/PPARΑ/VLCAD pathway, exerting beneficial effect in AF dogs.^112^ Although this study did not directly determine the contribution of AMPK by using AMPK inhibitor or knockout, it is reasonable to predict that AMPK occupies an important position in the disorders of lipid metabolism in AF because of the accepted theory that AMPK regulated energy homeostasis (Figure 4).^8^

## 4 DISCUSSION

DM is considered to be one of independent risk factors in the development of CVD.^113^ The prevalence rate of CVD is higher in diabetic adults than in non‐diabetic adults.^113^ Metformin, a widely used type II anti‐diabetic drug, could perform similar or even better cardiovascular protective effects than other drugs, such as glyburide and pioglitazone.^114^ This review summarized the current clinical evidence of metformin on cardiovascular system and described the underlying AMPK‐dependent molecular actions in several typical CVDs. We notice that considerable researchers hold the opinion that this cardiovascular benefit is independent of metformin's hypoglycaemic function.^115^, ^116^ However, this view does not fit the cases of all patients with various symptoms due to the complicated causes and risk factors of CVDs. Hypertension, physical inactivity, unhealthy diet, cholesterol and lipids, and stress are all classical cardiovascular risk factors, but are also common symptoms of diabetes.^117^ Diabetic cardiomyopathy (DCM) is a specific form of cardiomyopathy that occurs independently of other cardiac risk factors and is promoted by the long‐standing metabolic disorder of diabetes.^118^ In experimental DCM, metformin was able to attenuate high glucose‐induced apoptosis, inflammation and oxidative stress in cardiomyocytes, reinforcing the possibility that the protective effects of metformin in cardiomyocytes were highly related to its glycaemic control. Thus, it is extremely challenging to elucidate whether cardiovascular benefit of metformin is completely independent of its hypoglycaemic effects. As we can see from the existing clinical evidence, metformin was more efficient in T2DM patients to exert its cardio‐protective effects rather than in T1DM or non‐diabetic patients. It is reasonable to believe that cardiovascular benefit of metformin is partly due to the improvement in diabetes and partly due to its direct effects on endothelial cells or cardiomyocytes.

Although the molecular mechanisms regarding the cardiovascular benefits of metformin have not been fully clarified yet, it is generally accepted that AMPK activation is the predominant mechanism. Thus, this review highlights the protective effects of metformin mediated by AMPK activation. However, it is noteworthy to point out that there are also many proven non‐AMPK pathways in above CVDs.^119^ For instance, Prokinetin 2 (PK2), an small‐molecule secretory protein, participates in cardiomyocyte survival and angiogenesis by binding two agonist receptors, activin receptor 1 (pkr1) and activin receptor 2 (pkr2),^120^ and then could relieve I/R‐induced injury.^121^ According to this action, Yang et al proved that metformin activated PK2/PKR pathway to reduce apoptosis and improve cardiac function in DCM mice.^120^ In MI patients, the miR‐19a expression was decreased, leading to increased Acyl‐CoA synthetase long chain family member 1 (ACSL1) expression,^122^ a potential biomarker of acute MI risk.^123^ Peng et al utilized bioinformatics prediction and firstly revealed that metformin significantly inhibited apoptosis through miR‐19a/ACSL axis.^122^ Therefore, the cardio‐protective effects of metformin are achieved through both AMPK‐dependent and AMPK‐independent pathways with the domination of the former. Studies have demonstrated that AMPK knockout abolished the protective effect of metformin in some animal disease models.^42^, ^44^, ^52^, ^80^, ^109^

Apart from the functions of AMPK activation on apoptosis, oxidative stress, inflammation and metabolism that we emphasized here, novel discoveries regarding AMPK pathway and physiological process, including pyroptosis^124^, ^125^ and ferroptosis,^126^, ^127^ have emerged. These two novel cell death‐regulated forms play pivotal roles in the pathogenesis of various CVDs simultaneously.^128^, ^129^ However, whether positive effects of metformin are associated with pyroptosis or ferroptosis in endothelial cells and cardiomyocytes remains unclear. This would be a new research area, warranting future efforts to explore.

In addition to the hypoglycaemic function and cardio‐protective effects of metformin, it is noteworthy mentioning that scientists have found its pleiotropic effects on various diseases. A large number of cohort studies have shown that metformin is helpful for obesity treatment. It has been found that metformin could significantly reduce the weight of T2DM patients and non‐diabetic people.^130^, ^131^ The mechanism of the weight loss effect of metformin includes the commonly known effects on metabolism, and several newly explored effects such as on appetite regulation centres, intestinal microbiota and even related to ageing.^131^ Anti‐ageing is also a proven benefit of metformin. The attenuation of the characteristics of ageing can be achieved by regulating mitochondrial function, inhibiting cell ageing, etc.^132^ In addition, the use of metformin alone or in combination can induce ovulation in patients with polycystic ovary syndrome, but evidence to confirm the effect of reducing the abortion rate and increasing the live birth rate is still largely lacking.^133^ Notably, studies on metformin in the treatment of cancer have sprung over the years.^134^ A lot of clinical trials have found that long‐time exposure to metformin was associated with better survival in patients with tumours, such as lung cancer, bladder cancer and breast cancer.^135^ The action behind anti‐cancer effects of metformin is mainly a result of AMPK activation, mTOR inhibition, regulation of insulin and other AMPK‐independent pathways.^134^ For example, our group innovatively demonstrated that metformin targeted Clusterin to regulate lipogenesis and inhibit the growth of bladder cancer cells through SREBP‐1c/FASN axis.^136^ This study links metformin with Clusterin for the first time and further deepens the understanding of its anti‐cancer mechanism. Moreover, metformin has positive effect in the inflammatory skin disorders, in which its anti‐hyperglycaemic effect, NF‐kB inhibition and the anti‐hyperandrogenic effect played major roles.^137^ Some studies reported that metformin also played a significant role in cognitive impairment, mostly for nerve and nerve cell protection.^138^, ^139^ It normalized cell proliferation and neuroblast differentiation in the subgranular region of the hippocampal dentate gyrus of diabetic rats, and improved cognitive impairment caused by vitamin B12 deficiency.^139^ Based on above evidence, metformin has been called as the ‘aspirin’ of the 21st century.^140^

## 5 CONCLUSION AND PERSPECTIVE

Metformin displayed significant beneficial effects on cardiovascular health in both clinical trials and preclinical data integrally. In patients with DM, metformin was able to improve clinical cardiovascular outcomes with variable beneficial outcomes, depending on baseline characteristics of patients. It affords cardiovascular protective function mainly through reducing ECs and cardiomyocyte apoptosis, reducing oxidative stress, alleviating inflammation responses, improving mitochondrial function and regulating lipid homeostasis in animal models with CVD. The underlying molecular mechanisms are mainly metabolic transduction pathway with AMPK activation and regulation of its downstream effectors, such as eNOS, mTOR and PGC‐1α.

Despite both positive clinical evidence and preclinical evidence of metformin were highlighted here, existing clinical data need to be finely double determined while extending the preclinical evidence into clinical scenarios need more contributions of scientific and medical efforts. We should pay attention on the results of mentioned negative results in animal models and limited clinical outcomes in non‐diabetes patients. Currently, considerable studies of ongoing randomized controlled trials are conducted in non‐diabetic patients with CVDs, aiming to provide stronger evidence on the efficiency of metformin in cardiovascular system (NCT05093959, NCT05177588, NCT04625946, NCT03629340). Additionally, the safety profile of metformin still remains unclear.^141^ The incidence of lactic acidosis is minimal at recommended dosages, but it is severe in patients with deterioration of renal function, respiratory failure or exacerbation of heart failure.^142^ Whether metformin‐associated lactic acidosis is responsible for the elevated mortality rates in these patients is difficult to clarify due to worsening of underlying aetiology.^142^ Furthermore, metformin has been reported to have some unexpected hazards recently, such as birth defects in offspring^143^ and cognitive deficits^144^ in rodents, which need further efforts to confirm these results and amend the clinical guidelines regarding precisely defined indications and dosage.

In conclusion, these published literatures have indicated that AMPK activation is the predominant pathway for the positive effects of metformin on CVD and whether metformin can be utilized for CVD in clinical settings merits further investigation.

## AUTHOR CONTRIBUTIONS

**Yizhi Bu:** Conceptualization (lead); data curation (lead); formal analysis (lead); writing – original draft (lead); writing – review and editing (lead). **Mei Peng:** Conceptualization (supporting); formal analysis (supporting); methodology (supporting); validation (equal). **Xinyi Tang:** Conceptualization (supporting); data curation (equal); formal analysis (supporting); writing – original draft (supporting); writing – review and editing (supporting). **Xu Xu:** Conceptualization (supporting); data curation (equal); formal analysis (supporting); writing – original draft (supporting); writing – review and editing (supporting). **Yifeng Wu:** Data curation (supporting); writing – original draft (supporting); writing – review and editing (supporting). **Alex F. Chen:** Conceptualization (equal); formal analysis (equal); methodology (equal); project administration (equal); supervision (equal); validation (equal); visualization (equal). **Xiaoping Yang:** Conceptualization (lead); funding acquisition (lead); methodology (equal); project administration (lead); supervision (equal); validation (lead).

## CONFLICT OF INTEREST

Authors have read the journal's authorship agreement and policy on conflicts of interest and declare that they have none.

## Data Availability

No original data in this review.
